# Microbiology and Outcomes of Institutionalized Patients With Stroke-Associated Pneumonia: An Observational Cohort Study

**DOI:** 10.3389/fmicb.2021.720051

**Published:** 2021-12-03

**Authors:** Jie Zhao, Lei-qing Li, Ning-xin Zhen, Lin-lin Du, Hui Shan, Yang Yu, Zhao-cai Zhang, Wei Cui, Bao-ping Tian

**Affiliations:** Department of Critical Care Medicine, The Second Affiliated Hospital, Zhejiang University School of Medicine, Hangzhou, China

**Keywords:** ischemic stroke, hemorrhagic stroke, pneumonia, microbiology, outcomes

## Abstract

**Background:** The attributable mortality and microbial etiology of stroke-associated pneumonia (SAP) vary among different studies and were inconsistent.

**Purpose:** To determine the microbiology and outcomes of SAP in the lower respiratory tract (LRT) for patients with invasive mechanical ventilation (MV).

**Methods:** In this observational study, included patients were divided into SAP and non-SAP based on a comprehensive analysis of symptom, imaging, and laboratory results. Baseline characteristics, clinical characteristics, microbiology, and outcomes were recorded and evaluated.

**Results:** Of 200 patients, 42.5% developed SAP after the onset of stroke, and they had a lower proportion of non-smokers (*p* = 0.002), lower GCS score (*p* < 0.001), higher serum CRP (*p* < 0.001) at ICU admission, and a higher proportion of males (*p* < 0.001) and hypertension (*p* = 0.039) than patients with non-SAP. Gram-negative aerobic bacilli were the predominant organisms isolated (78.8%), followed by Gram-positive aerobic cocci (29.4%). The main pathogens included *K. pneumoniae*, *S. aureus*, *H. influenzae*, *A*. *baumannii*, *P. aeruginosa*, *E. aerogenes*, *Serratia marcescens*, and *Burkholderia cepacia*. SAP prolonged length of MV (*p* < 0.001), duration of ICU stay (*p* < 0.001) and hospital stay (*p* = 0.027), shortened MV-free days by 28 (*p* < 0.001), and caused elevated vasopressor application (*p* = 0.001) and 60-day mortality (*p* = 0.001). Logistic regression analysis suggested that patients with coma (*p* < 0.001) have a higher risk of developing SAP.

**Conclusion:** The microbiology of SAP is similar to early phase of HAP and VAP. SAP prolongs the duration of MV and length of ICU and hospital stays, but also markedly increases 60-day mortality.

## Background

Pneumonia occurring within 7 days of the onset of stroke was defined as SAP, and it was associated with prolonged hospital stays and poor prognosis (Hassan et al., 2006; Hannawi et al., 2013; Ferrer and Torres, 2018). Morbidity and attributable mortality of SAP vary among different studies, and the pathophysiology of SAP is mainly explained by aspiration and stroke-induced immunodepression (Hannawi et al., 2013). Clinically, the trachea, main bronchus, and bronchus at various levels in the lung are uniformly called lower respiratory tract (LRT), having the ability to eliminate microbes and purify inhaled gas. Risk factors included dysphagia, impaired consciousness, and ineffective cough reflex, as they impaired the ability of the LRT of eliminating microbes from oropharyngeal contents and so allow pathogens to enter the lung (DiBardino and Wunderink, 2015; Mandell and Niederman, 2019). Diverse communities of pulmonary microbiota have been discovered, thus dysbiosis was considered related to impaired pulmonary defenses (Wunderink and Waterer, 2017; Mandell and Niederman, 2019). Besides, the alternation of oral microbial diversity was not only observed in the murine pneumonia model, but also among the patients’ population of post-stroke and ventilation-associated pneumonia (VAP), inspiring new directions to investigate pathogenesis (Boaden et al., 2017; Chiu and Miller, 2019; Emonet et al., 2019; Mandell and Niederman, 2019; Morinaga et al., 2019). In recent studies, elevated bronchoalveolar lavage (BAL) amylase levels were associated with a high risk of aspiration and positive-culture, while serum procalcitonin (PCT) was associated with a prognosis of aspiration pneumonia (AP) (El-Solh et al., 2011; Weiss et al., 2013; DiBardino and Wunderink, 2015; Legriel et al., 2019; Mandell and Niederman, 2019). However, the diagnosis of SAP still relies on clinical features, as the lack of gold standard biomarkers. According to the current epidemiology of SAP, *Staphylococcus aureus (S. aureus), Klebsiella pneumoniae (K. pneumoniae), Pseudomonas aeruginosa (P. aeruginosa), Escherichia coli (E. coli), Streptococcus pneumoniae (S. pneumoniae)*, and *Haemophilus influenzae (H. influenzae)* are considered as major pathogens, which is of high similarity to the microbiology of hospital-acquired pneumonia (HAP) or community-acquired pneumonia (CAP) (Hassan et al., 2006; Chen et al., 2013; Hannawi et al., 2013; Mingmei et al., 2015; Lin et al., 2019). Clinically, it is common to apply mechanical ventilation (MV) on critically ill patients to maintain normal gas exchange, however, the mechanical force from MV could damage the normal airway barrier and the ability of the LRT of eliminating microbes. MV is a risk factor of SAP, and critically ill patients with MV are the population with a high risk of developing SAP. Besides, prophylactic antibiotic treatment (PAT) has different effects in decreasing morbidity and mortality owing to distinctive subtypes of pneumonia as well as patient population (Kalra et al., 2015; Wunderink and Waterer, 2017; Leone et al., 2018). Whether to apply PAT or not and the choice of antibiotics remain controversial. In summary, investigating the microbiology of SAP of critically ill patients with MV is of high significance. We conduct this observational study in order to identify microbiology in the LRT and clinical features on such a strictly stratified population, to further optimize the convergence of antibiotics and find significant medical indicators.

## Materials and Methods

### Design and Population

We conducted a single-center observational study in an intensive care unit (ICU) of a Second Affiliated Hospital of Zhejiang University between January 2020 and March 2021. The study complied with the current version of the Helsinki Declaration and appropriate clinical practice guidelines. The trial was approved by the ethics committee of our hospital (2019 Ethical Review No. 343), and was registered in the Chinese Clinical Trial Registry (ChiCTR2000028849). Written informed consent was obtained from all of the included patients or the next of kin. Criteria for enrollment included ICU inpatient older 18, stroke onset within 72 h, and invasive MV at least for 24 h. Acute ischemic stroke was defined as acute onset, focal neurological deficits or panfacial nerve dysfunction, presence of a responsible lesion on imaging or duration of symptoms or signs for at least 24 h, exclusion of non-vascular causes, and cerebral hemorrhage. Acute hemorrhagic stroke was defined as acute onset, focal neurological deficit symptoms often accompanied by headache, vomiting, elevated blood pressure, and varying degrees of disturbance of consciousness, head imaging showed bleeding lesions, and exclusion of non-vascular cerebral etiology. The diagnostic criteria of SAP follow the modified Center for Disease Control and Prevention (CDC) criteria: (1) At least one of the following: fever (> 38°C) with no other recognized cause; leukopenia (< 4,000 WBC/mm^3^) or leukocytosis (> 12,000 WBC/mm^3^); for adults ≥ 70 years old, altered mental status with no other recognized cause; (2) at least two of the following: new onset of purulent sputum, or change in the character of sputum over a 24 h period, or increased respiratory secretions, or increased suctioning requirements; new onset or worsening cough, or dyspnea, or tachypnea; rales, crackles, or bronchial breath sounds; worsening gas exchange; (3) chest radiographs with at least one of the following: New or progressive and persistent infiltrate, consolidation, or cavitation (Smith et al., 2015). Exclusion criteria were infectious diseases (including pneumonia) within 3 months before the onset of stroke, antibiotics use within 3 months, comorbidities such as chronic obstructive pulmonary disease (COPD), interstitial lung disease, lung tumor, atelectasis, pulmonary edema, pulmonary embolism and autoimmunity diseases, medical history of dysphagia or gastroesophageal reflux, pregnancy, pulmonary imaging did not accord with the manifestations of pneumonia after discussion by the research team, or unclear medical history. Participants could withdraw from the study if they decided not to continue to take part in the study, without any specific reason.

### Procedures

Patients who met the included criteria would be recruited in our study at ICU admission. All enrolled patients were followed prospectively and managed according to the following protocol. Characteristic such as age, sex, type of stroke, previous medical history, and time from onset of stroke to intubation as well as smoking status and baseline data at ICU admission including Glasgow Coma Scale (GCS) score, white cell count (WBC), PCT, and CRP were collected and recorded. Besides this, outcomes such as duration of MV, duration of MV-free days by 28, duration of ICU stay, duration of hospital stay, vasopressor during ICU stay, 60-day mortality, and with or without PAT were also observed and recorded. If the chest imaging examination indicated pulmonary inflammation at ICU admission, a specimen should be obtained within 2 h of ICU admission. Otherwise, continuous surveillance based on clinical symptoms was needed, and chest imaging examination would be applied to these patients if symptoms appeared within 48 h of invasive MV. Once positive imaging evidence supported inflammation, a sample should be obtained as soon as possible. Quantitative analysis of fiberscope or semi-quantitative analysis of suction in an endotracheal tube were used to collect specimens. The process of obtaining the specimen, storage in a sterile container, and sending to the laboratory should be finished within 2 h (at room temperature). All specimens should be processed as soon as possible to ensure the activity of pathogenic bacteria, and strictly follow standard inspection procedures such as inoculation, culture, smear, staining, and observation. The sputum samples were also inoculated in a fungal culture medium. The automatic bacterial identification system (Merieux VITEK 2 Compact) was used for strain identification. Empirical antibiotic therapy was started according to local epidemiology and the antibiotic regimen would be modified if needed based on the bacterial culture results. Two treating physicians independently evaluated patients’ status and made a diagnosis for SAP independently. If their diagnoses conflicted, a team discussion would be performed to determine whether the diagnosis is established. During the observational period, patients would be included in the SAP group once they met the diagnostic criteria for SAP within 48 h of invasive MV, otherwise, they would be categorized into the non-SAP group. Monitoting won’t be stopped until subjects were discharged from the hospital, or died. Besides, if a patient was applied an operation (craniotomy or stereotactic evacuation of intracerebral hematoma) and received antibiotics on the day of sugery before diagnosed as SAP, he would be regarded as a patient treated by prophylactic antibiotic therapy (PAT). Surgeons, clinical pharmacists and ICU physicians discussed and made the final decision of whether apply PAT or not.

### Statistical Analysis

Baseline characteristics and outcomes were analyzed and described as mean ± SD, median (Interquartile range, IQR), percentage as appropriate. Continuous variables were compared by Student’s *t*-test if they were normally distributed, otherwise, were compared by the Wilcoxon Mann-Whitney *U*-test. Pearson χ^2^-test or Fisher exact test were applied to categorical variables to compare differences between SAP and non-SAP. To investigate potential predictors for SAP, a multivariable Logistic regression analysis was conducted. Sensitivity analyses were performed by removing those patients who received PAT. All tests were 2-tailed and statistical significance was determined at an α a level of 0.05. Statistical analyses were performed with the SPSS 20.0.

## Results

### Comparison of Patients With Stroke-Associated Pneumonia and With Non-stroke-Associated Pneumonia

**FIGURE 1:**
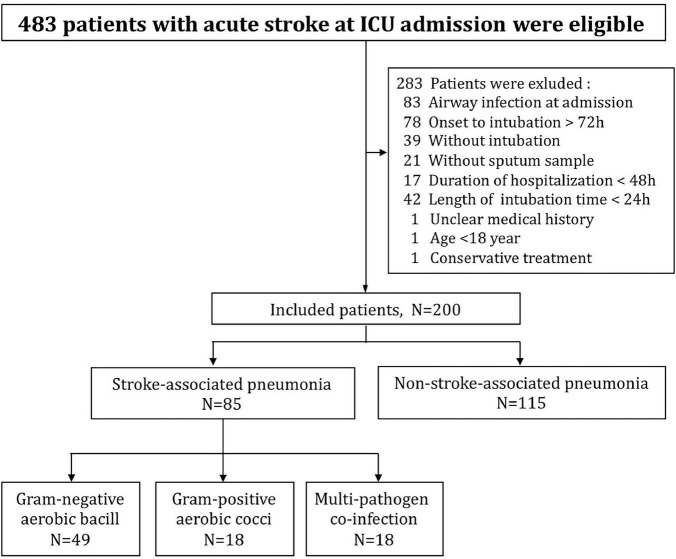
Flow diagram of specific process of the trial, illustrating the number of patients in each step and each group.

A total of 483 patients with acute stroke were admitted to ICU during the study, 283 were excluded according to inclusion and exclusion criteria. Finally, 200 patients were eligible for further analysis, and those were divided into two groups as SAP (*n* = 85) and non-SAP (*n* = 115) according to the microbiological results of sputum samples (Figure 1). No participants were withdrawn from the trial, and all included patients completed the study protocol and assessment of the main outcomes. Compared to patients without SAP, patients with SAP had lower GCS scores (median: 8 vs. 12.5, IQR: 5–10 vs. 8–15, ***p*** **< 0.001**), higher serum CRP scores (median: 12.9 vs. 5.3, IQR: 4.63–33.6 vs. 1.98–13.4, ***p*** **< 0.001**) at ICU admission, a higher proportion of males (63.5 vs. 36.5%, ***p*** **< 0.001**) and a higher proportion of hypertension (56.5 vs. 41.7%, ***p*** **= 0.039**). Besides, compared to the non-SAP group, more patients in the SAP group had a smoking history (47 vs. 23.5%, ***P*** **= 0.002**) (Table 1). No other baseline characteristics differed significantly between the two groups. For both groups, hemorrhagic stroke was the main type of stroke (90.6, 93.9%, respectively, *p* = 0.38) and over fifty percent of patients had the symptom of vomiting (56.5, 50.4%, respectively, *p* = 0.40) (Table 1).

**TABLE 1:** Baseline characteristics of the patients with and without microbiologically confirmed SAP at emergency admission.

Characteristics	SAP (*n* = 85)	Non-SAP (*n* = 115)	*P*
Age, mean ± SD (year)	55.9 ± 14.9	57.9 ± 13.3	0.33
Male, *n* (%)	54 (63.5)	42 (36.5)	< 0.001
Type of stroke			0.38
Ischemic stroke, *n* (%)	8 (9.4)	7 (6.1)	
Hemorrhagic stroke, *n* (%)	77 (90.6)	108 (93.9)	
Vomiting, *n* (%)	48 (56.5)	58 (50.4)	0.40
Time from onset of stroke to intubation, median (IQR) (hr)	19.5 (10-29.75)	24 (13-36)	0.14
At ICU admission			
GCS, median (IQR)	8 (5-10)	12.5 (8-15)	<0.001
White blood cell count, mean ± SD (×109/L)	12.1 ± 4.9	11.1 ± 3.73	0.09
C-reactive protein, median (IQR) (mg/L)	12.9 (4.63-33.6)	5.3 (1.98-13.4)	<0.001
Procalcitonin, median (IQR) (ng/mL)	0.13 (0.08-0.39)	0.11 (0.07-0.21)	0.14
**Comorbidities, *n* (%)**			
Hypertension	48 (56.5)	48 (41.7)	0.039
Heart disease	6 (7.1)	9 (7.8)	0.84
Diabetes mellitus	5 (5.9)	7 (6.1)	0.95
Renal disease	4 (4.7)	3 (2.6)	0.43
Liver disease	1 (1.2)	1 (1.7)	0.75
Smoking status			0.002
Current smoker, *n* (%)	28 (32.9)	19 (16.5)	
Ex-smoker, *n* (%)	12 (14.1)	8 (7)	
Non-smoker, *n* (%)	45 (52.9)	88 (76.5)	

*IQR, interquartile range; GCS, Glasgow Coma Scale; SD, standard deviation; hr, hour.*

### Clinical Characteristics of Patients With Microbiologically Confirmed Stroke-Associated Pneumonia

Of these 85 patients, 52.9% had an abnormal body temperature (median: 38.6, IQR: 38.2–38.85, °C), and 70.6% patients had leukocytosis or leukopenia (median: 12.3, IQR: 9.25–15, ×10^9^/L). Patients with purulent tracheobronchial aspirate occupied a higher proportion than those with non-purulent tracheobronchial aspirate (84.7% vs. 15.3%). According to chest radiographs, bilateral distribution (98.8%) and pleural effusion (87.1%) were important clinical characteristics, which appeared in almost all patients. Besides, serum CRP (median: 134, IQR: 59.95–187.7, mg/L) and serum PCT (median: 0.28, IQR: 0.1475–0.713, ng/mL) were found elevated during ICU stay for patients with SAP (Table 2). We performed sensitivity analysis by excluding those patients treated by PAT, and found consistent results in the remaining population (Supplementary Table 3).

**TABLE 2:** Clinical characteristics of the patients with microbiologically confirmed SAP.

Characteristics	SAP (*n* = 85)
Temperature ≤ 35.5°C or ≥ 38.5°C	
*n* (%)	45 (52.9)
Temperature, median (IQR) (°C)	38.6 (38.2-38.85)
Leukocytosis (WBC > 10 × 10^9^/L) or Leukopenia (WBC < 4 × 10^9^/L)	
*n* (%)	60 (70.6)
WBC, median (IQR) (×10^9^/L)	12.3 (9.25-15)
Tracheobronchial aspirate, *n* (%)	
Purulent	72 (84.7)
Non-purulent	13 (15.3)
≥2 criteria	
Chest radiograph, n (%)	
Pleural effusion	74 (87.1)
Bilateral	84 (98.8)
Multilobar	33 (38.8)
C-reactive protein, median (IQR) (mg/L)	134 (59.95-187.7)
Procalcitonin, median (IQR) (ng/mL)	0.28 (0.1475-0.713)

*IQR, interquartile range; n, number.*

### Microbial Etiology of Microbiologically Confirmed Stroke-Associated Pneumonia

**FIGURE 2:**
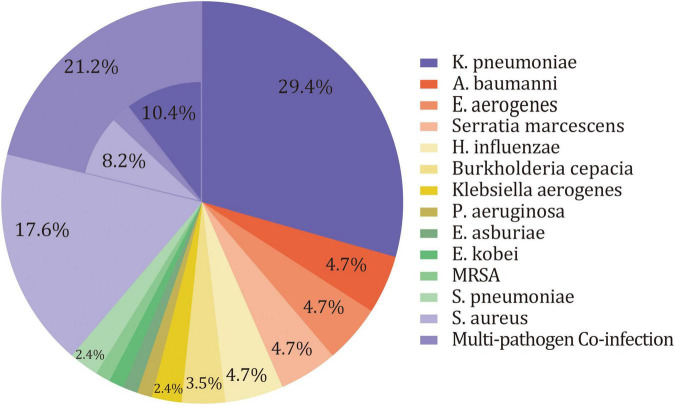
Pie chart of distribution of microbiology of SAP for critically ill patients.

Overall, 19 pathogens were isolated from deep sputum samplings for 85 patients in the SAP group. There were 67 patients who had one pathogen, of whom 49 were infected by Gram-negative aerobic bacilli, and 18 were infected by Gram-positive aerobic cocci. The remaining 18 patients had mixed infections, of which 16 patients had double infections and 2 patients had triple infections. Only one patient had anaerobe (*M. morganii*) in cultures. The remaining patients were infected by aerobic bacterium, and most of them were facultative anaerobes. Gram-negative aerobic bacilli were the predominant organisms isolated (78.8%), followed by Gram-positive aerobic cocci (29.4%). The majority of Gram-negative aerobic bacilli was *K. pneumoniae* (40%). Other Gram-negative aerobic bacilli included *Acinetobacter baumannii (A. baumannii*), *Enterobacter aerogenes (E. aerogenes), Serratia marcescens*, *H. influenzae*, *Burkholderia cepacian, Klebsiella aerogenes, P. aeruginosa, Enterobacter asburiae (E. asburiae)*, and *Enterobacter kobei (E. kobei).* As for Gram-positive aerobic cocci, *S. aureus predominated* (25.9%), followed by *S. pneumoniae* and *Methicillin-resistant S. aureus* (*MRSA*) (Table 3 and Figure 2). After excluding patients treated with PAT (*n* = 23), sensitivity analysis still suggested a similar microbiology of SAP in the remaining patients (Supplementary Table 4).

**TABLE 3:** Microbial etiology of SAP in LRT.

Microbiology	SAP (*n* = 85), *n* (%)
**Gram-negative aerobic bacilli**	49 (57.6)
*K. pneumoniae*	25 (29.4)
*A. baumannii*	4 (4.7)
*E. aerogenes*	4 (4.7)
*Serratia marcescens*	4 (4.7)
*H. influenzae*	4 (4.7)
*Burkholderia cepacia*	3 (3.5)
*Klebsiella aerogenes*	2 (2.4)
*P. aeruginosa*	1 (1.2)
*E. asburiae*	1 (1.2)
*E. kobei*	1 (1.2)
**Gram-positive aerobic cocci**	18 (21.2)
*S. aureus*	15 (17.6)
*S. pneumoniae*	2 (2.4)
*MRSA*	1 (1.2)
**Multi-pathogen co-infection**	18 (21.2)
Double infection	16 (18.8)
*K. pneumoniae* + *S. aureus*	3 (3.5)
*K. pneumoniae* + *H. influenzae*	2 (2.4)
*K. pneumoniae* + *P. aeruginosa*	1 (1.2)
*K. pneumoniae* + *M. morganii*	1 (1.2)
*K. pneumonia* + *E. coli*	1 (1.2)
*S. aureus* + *E. cloacae*	2 (2.4)
*S. aureus* + *H. influenzae*	1 (1.2)
*S. aureus* + *A. baumannii*	1 (1.2)
*H. influenzae* + *Branhamella catarrhalis*	1 (1.2)
*H. influenzae* + *E. coli*	1 (1.2)
*P. aeruginosa* + *Burkholderia cepacia*	1 (1.2)
*A. baumannii* + *Burkholderia cepacia*	1 (1.2)
Triple infection	2 (2.4)
*K. pneumoniae* + *P. aeruginosa* + *Carbapenem-resistant A. baumannii*	1 (1.2)
*P. aeruginosa* + *A. baumannii* + *Proteus mirabilis*	1 (1.2)

*K. pneumoniae, Klebsiella pneumoniae; A. baumannii, Acinetobacter baumannii; E. aerogenes, Enterobacter aerogenes; H. influenzae, Haemophilus influenzae; P. aeruginosa, Pseudomonas aeruginosa; E. asburiae, Enterobacter asburiae; E. kobei, Enterobacter kobei; S. aureus, Staphylococcus aureus; S. pneumoniae, Streptococcus pneumoniae; MRSA, Methicillin-resistant S. aureus; M. morganii, Morganella morganii; E. coli, Escherichia coli; E. cloacae, Enterobacter cloacae.*

### Prophylactic Antimicrobial Therapy of Post-stroke Patients With Stroke-Associated Pneumonia or Non-stroke-Associated Pneumonia

Of these 85 patients with SAP, 23 (27.1%) received PAT, 60 (70.6%) only received therapeutic antibiotic medication after SAP diagnosis. As for patients with non-SAP, 20 (17.4%) received PAT and 69 (60%) received therapeutic antibiotic medication (Supplementary Table 1). Antibiotics used included cefuroxime, piperacillin-tazobactam, clindamycin, cefotaxime, cefoperazone-sulbactam, levofloxacin, latamoxef, and ceftriaxone. Besides, the microbiology of patients with SAP and the corresponding antibiotics regimen were illustrated in Supplementary Table 2. The most common antibiotic used in pre-operative prophylactic application was cefuroxime.

### Outcome Comparison of Patients With Stroke-Associated Pneumonia and Non-stroke-Associated Pneumonia

Compared to patients with SAP (median: 160, IQR: 94.5–250, h), those without SAP spent less time on MV (median: 36, IQR: 15–139, ***p*** **< 0.001**). Consistently, patients without SAP (median: 26.5, IQR: 22.2–27.4, days) have more MV-free days by 28 than patients with SAP (median: 21.3, IQR: 17.6–24.1, ***p*** **< 0.001**). SAP prolonged duration of ICU stays (median: 10 vs. 3, IQR: 6–14 vs. 2–9, days, ***p*** **< 0.001**) and hospital stays (median: 14 vs. 11, IQR: 10–20 vs. 9–16, days, ***p*** **= 0.027**) compared with non-SAP. More patients with SAP (*n* = 25, 29.4%) received vasopressor during ICU stays than patients with non-SAP (*n* = 13, 11.3%, ***p*** **= 0.001**). In addition, significant difference in 60-day mortality was also found between patients with SAP (*n* = 18, 21.2%) and patients with non-SAP (*n* = 6, 5.2%, ***p*** **= 0.001**) (Table 4). Sensitivity analysis by excluding patients treated with PAT suggested that SAP (*n* = 62) had prolonged durations of MV, ICU stay, and hospital stay, and a higher 60-day mortality than non-SAP (*n* = 95) (Supplementary Table 5).

**TABLE 4:** Outcomes of the patients with microbiologically confirmed SAP compared with matched control.

Characteristics	SAP (*n* = 85)	Non-SAP (*n* = 115)	*P*
Duration of mechanical ventilation, median (IQR) (hr)	160 (94.5-250)	36 (15-139)	<0.001
Mechanical ventilation-free days by 28, median (IQR) (day)	21.3 (17.6-24.1)	26.5 (22.2-27.4)	<0.001
Duration of ICU stay, median (IQR) (day)	10 (6-14)	3 (2-9)	<0.001
Duration of hospital stay, median (IQR) (day)	14 (10-20)	11 (9-16)	0.027
Vasopressor during ICU stay, n%	25 (29.4)	13 (11.3)	0.001
60-day mortality, n%	18 (21.2)	6 (5.2)	0.001

*IQR, interquartile range; hr, hour.*

### The Potential Predictors for Stroke-Associated Pneumonia at Intensive Care Unit Admission

**FIGURE 3:**
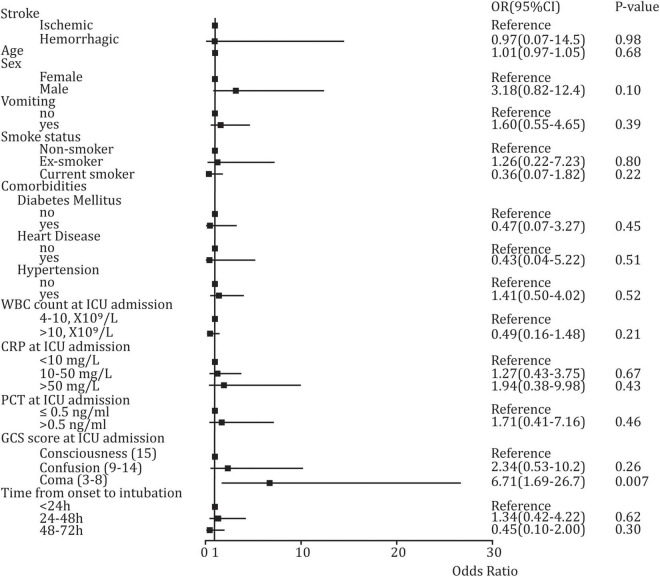
Forest plot of Logistic regression analysis, describing the potential predictors for SAP.

In univariate analysis, significant difference was found in such baseline variables as GCS score at ICU admission (***p*** **< 0.001**), CRP score at ICU admission (***p*** **< 0.001**), sex (***p*** **< 0.001**), hypertension (***p*** **= 0.039**), and smoking status (***p*** **= 0.002**) between SAP and non-SAP (Table 1). However, multivariable Logistic regression analysis suggested that only coma (GCS score: 3–8) was a high-risk factor for developing SAP (OR: 6.71, 95%CI: 1.69–26.7, ***p*** **= 0.007**), and a significant difference was not found in other variables (Figure 3).

## Discussion

Retrospective studies conducted by Josephson et al. (2010) and Kasuya et al. (2011) suggested that VAP was associated with prolonged duration of MV and duration of ICU stay, but not associated with increased mortality. We innovatively investigated the microbiology of SAP in LRT for post-stroke patients with invasive MV. In our study, the main attributed pathogens were *K. pneumoniae* (40%), *S. aureus* (25.9%), *H. influenzae*, *A*. *baumannii*, *P. aeruginosa, E. aerogenes*, *Serratia marcescens*, and *Burkholderia cepacia*, similar to the microbiology of culture-positive pneumonia for hospitalized neurologic patients and early onset VAP (Kalanuria et al., 2014; Nair and Niederman, 2015; Lee et al., 2019). Besides, a systemic review involving 7968 patients with acute stroke indicted that the commonly isolated organisms of SAP included *K. pneumoniae* (12.8%), *E. coli* (9%), *S. aureus* (10.1%), P. *aeruginosa* (6%), *A. baumanii* (4.6%), and *S. pneumoniae* (3.5%) (Kishore et al., 2018). The specific frequency for each pathogen was discrepant among different studies. This discrepancy might be attributed to the heterogeneity of epidemics in different regions, the heterogeneity of clinical environments, whether the patients were treated with invasive treatments or PAT, and so on. For instance, this systemic analysis pointed out that studies whose patients were at relatively higher risk, such as dysphagic or ICU admission, had a high proportion of Gram-negative aerobic bacilli and S. aureus, which is consistent with findings in our study (Kishore et al., 2018).

Critically ill patients have many risk factors of SAP, including hypertension, dysphagia, MV, male, a lower consciousness level, and bedridden (Liu et al., 2018). Univariate and multivariable analysis in our study indicated that a relatively lower consciousness level was an important high-risk factor for developing SAP. The majority of patients in our study were diagnosed with hemorrhagic stroke. However, ischemic stroke is the more common type of stroke, and most researchers have recruited patients from stroke units (ischemic stroke predominated) or directly selected patients with ischemic stroke (Warusevitane et al., 2015; Kalra et al., 2016; Kishore et al., 2018). Ischemic stroke and hemorrhagic stroke have a lot of differences including mechanism, symptoms, and prognosis. And whether these divergences would further impact SAP are still unclear. For example, stroke-induced immunodepression was found to be correlated with the mechanism and morbidity risk of SAP for patients with ischemic stroke (Hoffmann et al., 2017; Urra et al., 2017). However, whether immunodepression induced by hemorrhagic stroke is identical to ischemic stroke is still uncertain. Whether serum CRP and PCT could be used in diagnosing SAP and the differential diagnosis of SAP were still unclear (Smith et al., 2015). Improvements to diagnostic sensitivity and specificity need comprehensive consideration of symptoms and biomarkers. For afebrile patients with chest findings and leukocytosis or new radiological chest infiltrates, Kalra et al. (2019) highly recommended CRP ≥ 30 mg/L as a supplement to diagnosed SAP. This strategy remedied missed diagnoses owing to afebrile, to some extent. In our study, CRP at ICU admission was elevated mildly in the SAP group (12.9 vs. 5.3 mg/L, *p* < 0.001) compared to non-SAP, which might be a somatic response to acute stroke or correlate with stroke-induced immunodepression. The elevation of serum CRP in early phase might help recognize potential patients with SAP, which need more attention and further investigation. In recent studies, serum PCT levels were found to be significantly higher in patients with Gram-negative sepsis than in those with Gram-positive or fungal sepsis, suggesting a correlation between serum PCT levels and diagnostic etiological orientation (Li et al., 2016; Thomas-Rüddel et al., 2018). More studies should be performed to figure out whether serum PCT levels related with the type of pathogens for SAP or not. In addition, dynamic change process of biomarkers (e.g., CRP and PCT) reflect the progression and prognosis of the disease. PCT was considered to have higher diagnostic and prognostic accuracy of SAP than CRP in recent studies (Wang et al., 2016; Shi et al., 2021). Interestingly, PCT-guided antimicrobial strategies in ICU attracted a lot of attention, and PCT-guided cessation of antibiotics was found to reduce both antibiotic exposure and mortality (Huang et al., 2017; Lam et al., 2018). PCT might be a good indicator reflecting the progression of SAP and guiding clinical medication. More studies should be designed to clarify the details about the role of the serum CRP and PCT in SAP. In our study, patients with SAP had higher 60-day mortality than non-SAP (21.2 vs. 5.2%). Finlayson et al. (2011) reported higher 30-day mortality (39.5 vs. 10.3%) in SAP than non-SAP for patients with ischemic stroke, while Suda et al. (2018) also suggested higher 3-month mortality in stroke-associated infection than control (24.3 vs. 3.9%) for patients with acute stroke.

Current guidance discouraged applying PAT before SAP diagnosis established. However, some patients in our study were given PAT, as they indeed faced a high risk of infection and had a poor physical status. Practically, physicians still would choose PAT in clinical work, considering the infectious threat patients faced and the low probability of victory of anti-infection only relying on themselves. Whether PAT favors preventing infection and prognosis is a controversial topic, and several studies have been performed (Kalra et al., 2015; Westendorp et al., 2015; Shim and Wong, 2016; Smith et al., 2019). Westendorp et al. (2015) explored the positive effects of ceftriaxone on outcomes for patients with acute stroke, whereas Kalra et al. (2015) investigated whether prophylactic antibiotics could reduce pneumonia in post-stroke patients with dysphagia. The former found PAT could reduce the incidence of infection, while the latter illustrated that PAT did not reduce the frequency of pneumonia. The consistent conclusion in these two high-quality studies was that PAT did not improve functional outcomes at 3 months. The above findings suggested PAT was only capable of preventing urinary tract infections, not SAP (Meisel and Smith, 2015). In summary, no antibiotics have been found to have beneficial effects in preventing SAP so far. However, specific antibiotic protocols in each study succumbed to local hygiene policies and local empiric administration habits. Besides, type of antibiotics, time point of administration, dose, mode of administration, type of population will also impact the final effects of antibiotics. Furthermore, the drug resistance of the isolated germs was still uncertain, therefore antibiotic therapy remains empirical. Another point that attracted our attention is the neuroprotective effects of some antibiotics. Previous studies reported the neuroprotective effects of ceftriaxone and minocycline in mouse models with neurological injury (Thöne-Reineke et al., 2008; Yang et al., 2015; Dai et al., 2019). As we all know, neurovascular injury is the primary etiology of SAP, and so antibiotics would be beneficial in the early phase of stoke if they have neuroprotective effects. In summary, it is insufficient to totally negate the effects of PAT in SAP just depending on evidence from current studies.

There are some limitations to our study. Firstly, our study is a single-center observational cohort study and the sample size was relatively small. Since the microbiology of SAP is associated with local epidemiology, our findings need to be further validated in a larger cohort based on multi-center cooperation. Secondly, our study only focused on patients’ short-term outcomes such as duration of ICU stays and 60-day mortality. Long-term follow-up should be performed. Thirdly, as microbiologically confirmed bacterial pneumonia was our focus, the determination of infection strictly followed the results of sputum bacterial culture, and virus detection was not performed in our study. It would be better when broader pathogen (e.g., bacterium and viruses) distribution and frequency were figured out. Finally, some patients received PAT since they had a high risk of infection and serious status, which might impact the results of bacterial culture. The main antibiotic in our study was cefuroxime, which is a broad-spectrum antibiotic. Thus, the actual morbidity of SAP might be higher than the results obtained in our study.

## Conclusion

Overall, this is the first observational cohort study to investigate the microbiology of SAP in LRT for critically post-stroke patients treated with invasive MV. Those patients are at high risk of developing SAP, especially patients with hypertension and relatively poor status of consciousness. We found the main pathogens responsible for SAP in LRT were *K. pneumoniae*, *S. aureus*, *H. influenzae*, *A*. *baumanni*, *P. aeruginosa, E. aerogenes*, *Serratia marcescens*, *Burkholderia cepacian*, etc., which is similar to the microbiology of early phase HAP and VAP. Besides, SAP would not only significantly prolong the duration of MV, and the length of ICU stay and hospital stay, but also increase vasopressor during ICU stays as well as mortality.

## Data Availability Statement

The datasets presented in this study can be found in online repositories. The names of the repository/repositories and accession number(s) can be found in the article/Supplementary Material.

## Ethics Statement

The studies involving human participants were reviewed and approved by the Second Affiliated Hospital, Zhejiang University School of Medicine. The patients/participants provided their written informed consent to participate in this study. Written informed consent was obtained from the individual(s) for the publication of any potentially identifiable images or data included in this article.

## Author Contributions

B-PT and L-QL contributed to the conception and design of this study. JZ and N-XZ acquired the data. L-LD, HS, and WC analyzed and interpreted the data. JZ and B-PT drafted the manuscript. JZ, L-QL, and YY conducted the statistical analysis. B-PT, Z-CZ, and WC supervised the study. All authors reviewed drafts of the manuscript and approved the final version.

## Conflict of Interest

The authors declare that the research was conducted in the absence of any commercial or financial relationships that could be construed as a potential conflict of interest.

## Publisher’s Note

All claims expressed in this article are solely those of the authors and do not necessarily represent those of their affiliated organizations, or those of the publisher, the editors and the reviewers. Any product that may be evaluated in this article, or claim that may be made by its manufacturer, is not guaranteed or endorsed by the publisher.
