# Supplementary material for: Microbiology and Outcomes of Institutionalized Patients With Stroke-Associated Pneumonia: An Observational Cohort Study
Source: Front Microbiol. 2021 Dec 3;12:720051. doi: 10.3389/fmicb.2021.720051 (PMC8678279; doi:10.3389/fmicb.2021.720051)
Supplement: Supplementary file 1 [file Data_Sheet_1.zip › Table 1 (1).DOCX]

**Online supplemental Table S1**

Antibiotic use for included patients on the day of surgery when they haven’t been diagnosed with SAP.

| Antibiotic use, n (%) | | SAP (n=85) | Non-SAP(n=115) |
| --- | --- | --- | --- |
| Only Therapeutic use after SAP diagnosis | | 60 (70.6) | 69 (60) |
| Cefuroxime | | 35 (41.2) | 50 (43.5) |
| Piperacillin-Tazobactam | | 17 (20) | 8 (7) |
| Clindamycin | | 3 (3.5) | 6 (5.2) |
| Cefuroxime + Piperacillin-Tazobactam | | 4 (4.7) | 1 (0.9) |
| Cefotaxime | | 1 (1.2) | 0 (0) |
| Cefoperazone-Sulbactam | | 0 (0) | 2 (1.7) |
| Latamoxef | | 0 (0) | 1 (0.9) |
| Levofloxacin | | 0 (0) | 1 (0.9) |
| With Prophylactic Antibiotic Therapy (PAT) | | 23 (27.1) | 20 (17.4) |
| PAT | Therapeutic |  |  |
| Cefuroxime | Cefuroxime | 14 (16.5) | 16 (13.9) |
| Cefuroxime | Latamoxef | 0 (0) | 2 (1.7) |
| Cefuroxime | Piperacillin-Tazobactam | 2 (2.4) | 0 (0) |
| Cefoperazone-Sulbactam | Piperacillin-Tazobactam | 0 (0) | 1 (1.7) |
| Clindamycin | Clindamycin | 0 (0) | 1 (1.7) |
| Ceftriaxone | Piperacillin-Tazobactam | 1 (1.2) | 0 (0) |
| Cefuroxime | Cefuroxime + Piperacillin-Tazobactam | 6 (7.1) | 0 (0) |
| Without antibiotic use | | 2 (2.4) | 26 (22.6) |

PAT = prophylactic antibiotic therapy; SAP = stroke-associated pneumonia.

**Online supplemental Table S2**

Microbiology of SAP and the corresponding antibiotic administration

| **Antibiotic use** | | **Microbiology** |
| --- | --- | --- |
| **Only Therapeutic use after SAP diagnosis** | | |
| Cefuroxime | | *K. pneumoniae, A. baumannii, S. aureus, H. influenzae,*  *M. morganii, P. aeruginosa, E. cloacae, MRSA, Serratia marcescens, Branhamella catarrhalis, Klebsiella aerogenes, E. asburiae, E. kobei, E. aerogenes, Burkholderia cepacian* |
| Piperacillin-Tazobactam | | *K. pneumoniae, S. aureus, P. aeruginosa, A. baumannii,*  *E. aerogenes, Proteus mirabilis, Burkholderia cepacian, H. influenzae* |
| Clindamycin | | *K. pneumoniae, A. baumannii, S. aureus,* |
| Cefuroxime **+** Piperacillin-Tazobactam | | *S. aureus, A. baumannii, E. cloacae,* |
| Cefotaxime | | *K. pneumoniae* |
| **With Prophylactic Antibiotic Therapy (PAT)** | | |
| **PAT** | **Therapeutic** |  |
| Cefuroxime | Cefuroxime | *K. pneumoniae, A. baumannii, S. aureus, H. influenzae,*  *P. aeruginosa, Burkholderia cepacian, Serratia marcescens, Carbapenem-resistant A. baumannii, S. pneumoniae, E. aerogenes* |
| Cefuroxime | Piperacillin-Tazobactam | *K. pneumoniae, E. coli, E. aerogenes* |
| Ceftriaxone | Piperacillin-Tazobactam | *K. pneumoniae* |
| Cefuroxime | Cefuroxime +  Piperacillin-Tazobactam | *K. pneumoniae, H. influenzae, P. aeruginosa, S. aureus, Burkholderia cepacian, S. pneumoniae, E. coli* |
| **Without antibiotic use** | | *K. pneumoniae* |

PAT = prophylactic antibiotic therapy; SAP = stroke-associated pneumonia

**Online supplemental Table S3**

Clinical characteristics of the patients with microbiologically confirmed SAP (for those without PAT)

| **Characteristics** | **SAP (n=62)** |
| --- | --- |
| Temperature ≤35.5 ℃ or ≥38.5 ℃ |  |
| n (%) | 34 (54.8) |
| Temperature, median (IQR) (℃), | 38.6 (38.4-38.9) |
| Leukocytosis (WBC>10X10^9^/L) or Leukopenia  (WBC<4X10^9^/L) |  |
| n (%) | 44 (71.0) |
| WBC , median (IQR) (X10^9^/L) | 12.9 (9.33-15.8) |
| Tracheobronchial aspirate, n (%) |  |
| Purulent | 52 (83.9) |
| Non-purulent | 10 (16.1) |
| ≥ 2 criteria |  |
| Chest radiograph, n (%) |  |
| Pleural effusion | 48 (77.4) |
| Bilateral | 60 (96.8) |
| Multilobar | 22 (35.5) |
| C-reactive protein, median (IQR) (mg/L) | 130.9 (62.6-178.4) |
| Procalcitonin, median(IQR) (ng/mL) | 0.24 (0.16-0.58) |

PAT = Prophylactic Antibiotic Therapy, IQR = interquartile range, n=number

**Online supplemental Table S4**

Microbial etiology of SAP in LRT for patients without PAT

| **Microbiology** | **SAP (n=62), n(%)** |
| --- | --- |
| **Gram-negative aerobic bacilli** | **35 (56.5)** |
| *K. pneumoniae* | 18 (29) |
| *A. baumannii* | 3 (4.8) |
| *E. aerogenes* | 2 (3.2) |
| *Serratia marcescens* | 3 (4.8) |
| *H. influenzae* | 2 (3.2) |
| *Burkholderia cepacia* | 2 (3.2) |
| *Klebsiella aerogenes* | 2 (3.2) |
| *P. aeruginosa* | 1 (1.6) |
| *E. asburiae* | 1 (1.6) |
| *E. kobei* | 1 (1.6) |
| **Gram-positive aerobic cocci** | **14 (22.6)** |
| *S.aureus* | 13 (21.0) |
| *MRSA* | 1 (1.6) |
| ***Multi-pathogen co-infection*** | **13 (21.0)** |
| Double infection | 12 (19.4) |
| *K. pneumoniae* + *S. aureus* | 3 (4.8) |
| *K. pneumoniae* + *H. influenzae* | 2 (3.2) |
| *K. pneumoniae* + *P. aeruginosa* | 1 (1.6) |
| *K. pneumoniae* + *M.morganii* | 1 (1.6) |
| *S. aureus* + *E. cloacae* | 2 (3.2) |
| *S. aureus* + *A. baumannii* | 1 (1.6) |
| *H. influenzae* + *Branhamella catarrhalis* | 1 (1.6) |
| *A. baumannii* + *Burkholderia cepacia* | 1 (1.6) |
| Triple infection | 1 (1.6) |
| *P. aeruginosa* + *A. baumannii* + *Proteus mirabilis* | 1 (1.6) |

PAT = Prophylactic Antibiotic Therapy, *K. pneumoniae = Klebsiella pneumoniae; A. baumannii = Acinetobacter baumannii; E. aerogenes = Enterobacter aerogenes; H. influenzae = Haemophilus influenzae; P. aeruginosa = Pseudomonas aeruginosa; E. asburiae = Enterobacter asburiae; E. kobei = Enterobacter kobei; S.aureus = Staphylococcus aureus; MRSA = Methicillin-resistant S. aureus; M.morganii = Morganella morganii; E. cloacae = Enterobacter cloacae;*

**Online supplemental Table S5**

Table S5. Outcomes of the patients with microbiologically confirmed SAP compared with matched control (for those patients without PAT)

| **Characteristics** | **SAP**  **(n=62)** | **Non-SAP**  **(n=95)** | **P** |
| --- | --- | --- | --- |
| Duration of mechanical ventilation, median (IQR) (hr) | 155 (95.5-244) | 23 (13-72) | **<0.001** |
| Mechanical ventilation-free days by 28, median (IQR) (day) | 21.5 (17.8-24) | 27 (25-27) | **<0.001** |
| Duration of ICU stay, median (IQR) (day) | 11 (6-16) | 3 (2-6) | **<0.001** |
| Duration of hospital stay, median (IQR) (day) | 14.5 (10-21.3) | 11 (8-15) | **0.001** |
| Vasopressor during ICU stay, n% | 21 (33.9) | 13 (13.7) | **0.003** |
| 60-day mortality, n% | 14 (22.6) | 5 (5.3) | **0.001** |

PAT = Prophylactic Antibiotic Therapy, IQR = interquartile range, hr = hour
